# Invasive Pulmonary Aspergillosis and Nocardia Pneumonia in a Pediatric Patient With Chronic Lung Disease: A Case Report

**DOI:** 10.1155/crpe/6659641

**Published:** 2025-07-17

**Authors:** Ahmed H. Ali, Mahmoud Kamal, Mohammed E. Elyan

**Affiliations:** Pediatric Critical Care, Al Qassimi Women and Children Hospital, Emirates Health Services, Sharjah, UAE

## Abstract

This case illustrates the susceptibility of pediatric patients with chronic lung disease, here, a 3-year-old male with structural airway injury and impaired mucociliary clearance from recurrent aspiration (due to Chiari II malformation and spinal dysgenesis), to life-threatening polymicrobial co-infections, even without classic immunodeficiency. The child was admitted with acute respiratory failure and sepsis; imaging demonstrated necrotizing pneumonia and pneumatoceles; bronchoalveolar lavage confirmed ∗*Aspergillus*∗ galactomannan positivity and ∗*Nocardia*∗ species, warranting broad antimicrobial therapy and mechanical ventilation. Diagnosis of invasive aspergillosis and treatment initiation were delayed; despite therapy, he developed multiorgan failure and died. This highlights how chronic lung disease predisposes children to catastrophic fungal–bacterial synergies and reinforces the value of early bronchoscopy for targeted treatment. Advanced disease with structural damage portends poor outcomes, underscoring the need for enhanced surveillance in this high-risk cohort.

## 1. Introduction

Chronic lung disease of infancy predisposes children to opportunistic infections due to impaired mucociliary clearance and chronic pulmonary inflammation. This risk is amplified when the disease stems from recurrent aspiration or structural airway injury [1]. While immunocompromised states (e.g., transplantation and HIV) are established risks for infections such as aspergillosis or nocardiosis, structural lung pathology alone is sufficient to classify the patient as an at-risk host [2]. We describe a fatal case of co-existent pulmonary aspergillosis and nocardiosis in a child with chronic lung disease secondary to neurogenic dysphagia. This case underscores how structural lung injury, rather than classic immunodeficiency, can drive catastrophic polymicrobial infections.

## 2. Case Presentation

A 3-year-old male with chronic lung disease secondary to recurrent aspiration (neurogenic dysphagia due to Chiari II malformation and spinal dysgenesis) presented to the emergency department with acute respiratory distress. His medical history included the following:

- Hydrocephalus dependent on a VP shunt
- Vesicoureteral reflux Grade V treated with intermittent catheterization
- Chronic lung disease with frequent hospitalizations for pneumonia

**Figure 1:**
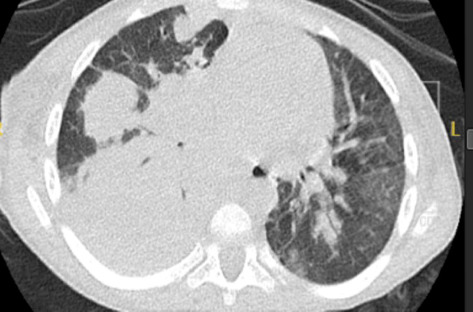
Chest CT showing severe necrotizing pneumonia and pneumatocele formation.

The child presented with acute respiratory distress, hypoxia (SpO_2_ 88%), labored breathing, and intercostal retractions. Chest X-ray showed right upper and lower lobe consolidation. Empiric therapy with ceftriaxone, vancomycin, and oseltamivir was initiated. Laboratory results revealed significant leukocytosis (WBC: 38.65 × 10^3^/μL), markedly elevated CRP (302 mg/L), and normal immunoglobulin levels (IgA: 194 mg/dL, IgG: 1010 mg/dL, and IgM: 164 mg/dL). His hypoxemia worsened after 48 h despite high-flow oxygen. CT chest performed at this time showed bilateral consolidations, ground-glass opacities, and pneumatoceles, consistent with fungal or necrotizing bacterial pneumonia. Concurrently, as shown in Figure 1, significant anemia (Hb 6.8 g/dL) developed, delaying bronchoscopy and necessitating blood transfusions.

By Day 6 of admission, he progressed to respiratory failure requiring invasive mechanical ventilation. Chest ultrasound performed during this period revealed right lung collapse, prompting urgent tube thoracotomy.

Bronchoscopy was subsequently performed once the anemia was corrected. On the tenth day of admission, bronchoalveolar lavage (BAL) results returned positive for *Aspergillus* galactomannan, and *Nocardia* species were isolated. Targeted antifungal therapy with caspofungin and antibacterial therapy with trimethoprim-sulfamethoxazole (TMP-SMX) were immediately initiated.

Despite maximal respiratory support (invasive mechanical ventilation) and targeted antimicrobial therapy, his condition deteriorated further, evolving into refractory multiorgan failure (MOF). He developed profound and refractory hypotension, unresponsive to aggressive fluid resuscitation, requiring escalating doses of multiple intravenous vasopressors (e.g., norepinephrine and vasopressin) to maintain perfusion, indicating cardiovascular failure. Concurrently, he became oliguric, progressing to anuria, with biochemical evidence of severe acute kidney injury (rising serum creatinine and BUN), necessitating the initiation of renal replacement therapy (RRT) (continuous venovenous hemodialysis or hemofiltration, CVVHD/CVVH) due to renal failure. His respiratory failure remained severe, with persistent hypoxia and hypercapnia despite maximal ventilator settings (high FiO2 and high PEEP). The persistent, marked leukocytosis (WBC: 38.65 × 10^3^/μL) and extremely elevated CRP (302 mg/L) signaled uncontrolled sepsis and overwhelming systemic inflammation, contributing to a state of persistent hematologic/inflammatory dysregulation. This sequential and progressive failure of the respiratory, cardiovascular, renal, and hematologic/inflammatory systems defines refractory MOF.

In the final stages, he was dependent on maximal life support: invasive mechanical ventilation for refractory respiratory failure, high-dose multiple vasopressors for profound shock, and continuous RRT for anuric renal failure. Despite all interventions, including broad-spectrum and targeted antimicrobials/antifungals, mechanical ventilation, vasopressors, RRT, and prior transfusions, his MOF proved irreversible. He died due to complications of refractory septic shock and overwhelming pneumonia (*Aspergillus* and *Nocardia* co-infection), superimposed on his severe underlying chronic lung disease and complex comorbidities (neurogenic dysphagia, Chiari II malformation, spinal dysgenesis, VP shunt–dependent hydrocephalus, and VUR Grade V).

## 3. Discussion

The *Nocardia* spp. (partially acid-fast Gram-positive bacteria) and *Aspergillus* spp. (angioinvasive fungi) are opportunistic pathogens usually found in patients with immunocompromising conditions [3, 4]. With a dynamic molecular taxonomy, identification of *Nocardia* can be difficult, and *Aspergillus* can grow in damaged lungs through inhalation of airborne conidia. The patient's chronic lung disease from recurrent aspiration due to neurogenic dysphagia resulted in structural damage (e.g., bronchiectasis and pneumatoceles), favorable to pathogen colonization [4]. Neurogenic bladder, recurrent UTIs, and VP shunt revisions led to systemic inflammation, while prolonged use of antibiotics disrupted microbiota, leading to fungal overgrowth [1, 2, 4]. Diagnostic pitfalls included clinical–radiologic mimicry of bacterial pneumonia (e.g., fever, consolidations, and pneumatoceles) [4], nondiagnostic imaging features such as “halo sign” and cavitary lesions [5], and procedural delay (e.g., BAL on Day 9 due to anemia) [3–5]. Therapeutic challenges ensued with renal failure requiring caspofungin instead of voriconazole (first-line antifungal with mandatory drug monitoring) and fluid overload hindering TMP-SMX pharmacokinetics [3, 6]. Recurrent infections, an immune response, or chronic inflammation further diminished macrophage/T-cell responses, resulting in further therapeutic ineffectiveness [1, 7].

Although pediatric co-infections of *Aspergillus* and *Nocardia* are rarely described, similar patterns have been observed in structurally injured but immunocompetent lungs. For instance, Poovazhagi et al. [4] reported a case of co-infection with *Nocardia* and *Aspergillus* in a child following submersion injury, where structural lung damage allowed polymicrobial invasion in the absence of classic immunosuppression. The authors emphasized that radiologic signs may be nonspecific in children, and early antifungal therapy should be initiated even before culture confirmation to avoid delays that may increase mortality (Poovazhagi et al. [4], invasive aspergillosis and nocardial pneumonia in an immunocompetent child). This aligns with our case, where delayed bronchoscopy and nonspecific imaging findings contributed to late diagnosis and poor outcome, reinforcing that chronic or acquired lung injury can predispose to opportunistic infections typically associated with immunocompromised states.

Compared to the sparse literature, pediatric nocardiosis mortality with MOF is > 50%, significantly higher than adult rates of 14%–40%, particularly in virtually compromised hosts (e.g., cystic fibrosis) [4, 6]. The patient's predisposition came not from classical immunodeficiency but rather (as in patients with chronic obstructive pulmonary disease [COPD]) from chronic lung disease (repeated aspiration and bronchiectasis) acting as a risk factor independent of immune status [4, 6]. Unlike many immunocompromised cohorts (e.g., transplant recipients), this patient had no extreme classical risks (HIV and diabetes), exemplifying how anatomical defects and chronic inflammation mimic immunosuppression [6, 7]. Key insights include the need for early bronchoscopy even during instability, renal-augmented tailoring of antimicrobials (e.g., caspofungin over voriconazole) [8, 9], and/or antifungal/antibacterial prophylaxis in chronic lung disease. Providers need to understand that structural insults increase infection susceptibility in a manner not dissimilar to classical methods of immune compromise, and that awareness and proactive management are required for vigilant surveillance [1, 2, 4, 6–10].

## 4. Conclusion

This case demonstrates the lethal synergy between pulmonary aspergillosis and nocardiosis in a 3-year-old with chronic lung disease from neurogenic dysphagia (Chiari II malformation). Crucially, the fatal co-infection arose not from classic immunodeficiency but from structural airway damage, recurrent aspiration leading to bronchiectasis and pneumatoceles, creating a permissive niche for opportunistic pathogens. Chronic inflammation (driven by neurogenic bladder, recurrent UTIs, and VP shunt revisions) and antibiotic-induced dysbiosis further disrupted local immunity, enabling *Aspergillus* and *Nocardia* colonization and invasion.

Critical barriers to survival emerged at multiple junctures:

Diagnostic delay from nonspecific symptoms (fever and cough) and imaging overlap (consolidations masking fungal/nocardial features).

Procedural bottlenecks, most notably, severe anemia delayed BAL until Day 10, by which time bilateral necrotizing pneumonia was advanced.

Therapeutic limitations were imposed by renal failure, forcing suboptimal antifungal selection (caspofungin over voriconazole) and impaired TMP-SMX clearance.

Despite aggressive dual-pathogen therapy and maximal organ support (mechanical ventilation, vasopressors, and RRT), rapid progression to refractory MOF ensued, underscoring the devastating virulence of fungal–bacterial co-infection in injured lungs. This outcome aligns with reported mortality > 50% in pediatric nocardiosis with MOF, particularly in structurally compromised hosts.

Three imperative lessons emerge as follows:

  Structural lung injury alone confers immunodeficiency-equivalent risk. Providers must suspect atypical pathogens (*Aspergillus* and *Nocardia*) early in children with aspiration-related lung damage, even without classic immunocompromise.
  Diagnostic urgency outweighs instability. Bronchoscopy/BAL and galactomannan testing should be expedited despite critical illness; mitigate barriers (e.g., transfuse for anemia) immediately.
  Prophylaxis deserves consideration in high-risk patients (e.g., chronic aspiration with bronchiectasis). Antifungal or antinocardial prevention may avert catastrophe where lung architecture is irrevocably damaged.
  This case redefines vulnerability: Anatomical defects and chronic inflammation can mimic traditional immunosuppression, demanding equally vigilant surveillance and tailored preemptive strategies. For children with neurogenic dysphagia and lung injury, proactive management of infection risk is not supportive care, it is a lifesaving intervention.

## Data Availability

The data that support the findings of this study are available from the corresponding author upon reasonable request.
